# *Lactobacillus crispatus* KBL693 alleviates atopic dermatitis symptoms through immune modulation

**DOI:** 10.71150/jm.2509005

**Published:** 2025-10-31

**Authors:** Seokcheon Song, Jun-Hyeong Kim, Sung Jae Jang, Eun Jung Jo, Sang Kyun Lim, GwangPyo Ko

**Affiliations:** 1 Department of Environmental Health Sciences, Graduate School of Public Health, Seoul National University, Seoul 08826, Republic of Korea; 2 KoBioLabs, Inc., Seoul 08826, Republic of Korea; 3 Institute of Health and Environment, Seoul National University, Seoul 08826, Republic of Korea; 4 N-Bio, Seoul National University, Seoul 08826, Republic of Korea

**Keywords:** atopic dermatitis, probiotics, *Lactobacillus crispatus*, gut-skin axis, immune response

## Abstract

Atopic dermatitis (AD) is a widespread inflammatory skin condition that affects the population worldwide. Given the implication of microbiota in AD pathogenesis, we investigated whether human-derived *Lactobacillus* strains could modulate AD. In this study, we identified *Lactobacillus crispatus* KBL693 as a probiotic candidate for AD treatment. In vitro, KBL693 suppressed mast cell degranulation and IL-4 production by T cells, suggesting its ability to attenuate key type 2 immune responses. Consistent outcomes were observed in a murine AD model, where oral administration of KBL693 alleviated disease symptoms and reduced hallmark type 2 immune markers, including plasma IgE as well as IL-4, IL-5, and IL-13 levels in skin lesions. In addition to downregulating these AD-associated immune responses, KBL693 promoted regulatory T cell (Treg) expansion in mesenteric lymph nodes, indicating its potential to restore immune balance. Collectively, these findings highlight the therapeutic potential of KBL693 for AD through enhancement of Tregs and suppression of type 2 immune responses.

## Introduction

Atopic dermatitis (AD) is a widespread inflammatory skin condition that affects the population worldwide (Asher et al., 2006; Deckers et al., 2012; Weidinger et al., 2018). Type 2 immune responses and mast cells are pivotal in the pathogenesis of AD (Akdis et al., 2020; Gandhi et al., 2016; Langan et al., 2020). Upon exposure to allergens, dendritic cells (DCs) capture, process, and present them to naïve CD4^+^ T cells, driving their polarization toward Th2 that produce IL-4. This cytokine promotes antibody class switching in B cells, resulting in IgE production. The IgE antibodies then engage FcεRI on mast cells, heightening their responsiveness to allergens. Allergen recognition by IgE on mast cells triggers intracellular signaling cascades that result in degranulation and the secretion of the contents in the granules, like histamine, tryptase, and β-hexosaminidase, ultimately driving allergic inflammation and disease manifestation. In addition to IL-4, IL-5, and IL-13 are central in recruiting eosinophils, stimulating mast cells, and promoting keratinocyte remodeling.

Human gut harbors 10–100 trillion live microorganisms, including bacteria, fungi, archaea, and protozoa, which together constitute the gut microbiota (Turnbaugh et al., 2007). Microbiota interact continuously and intimately with the host and influence its health, metabolism, and immune system (Belkaid and Hand, 2014; Lynch and Pedersen, 2016; Turnbaugh et al., 2006). Early-life colonization through vaginal delivery, breastfeeding, and environmental exposures profoundly shapes the gut microbial community and host immunity (Bogaert et al., 2023; McCauley et al., 2022; Vatanen et al., 2022). At birth, maternal vaginal bacteria are transmitted to the infant and facilitate the initial gut microbiota formation, thereby promoting immune tolerance and decreasing the allergic disease risk (McCauley et al., 2022). Consistently, in a mouse model, newborn pups delivered by cesarean section were orally administered human vaginal microbiota at birth, and this inoculation was shown to influence their health outcomes (Jašarević et al., 2021). The “Old friends” hypothesis proposes that reduced microbial exposure in developed societies contributes to the rising prevalence of allergic and autoimmune diseases by impairing immune education (Rook et al., 2013).

Accumulating evidence indicates that gut microbiota are critically involved in allergic diseases, including atopic dermatitis (Petersen et al., 2019; Russell et al., 2012; Wesemann and Nagler, 2016). For instance, neonatal gut microbial community types associated with an increased atopy risk were reported to influence T cell differentiation through microbial metabolites (Fujimura et al., 2016). Furthermore, intestinal short-chain fatty acids were shown to strengthen the skin barrier function by stimulating keratinocyte metabolic activity and maturation (Trompette et al., 2022). Together, these studies point to gut microbiota as an attractive focus for intervention aimed at controlling allergic disorders such as atopic dermatitis.

Probiotics, defined as live microorganisms that confer health benefits to the host (Hill et al., 2014; Kim et al., 2023; Sanders et al., 2019; Wang et al., 2023), have been explored as modulators of the gut microbiota and immune responses. Oral administration of various probiotic strains, such as *Lactobacillus acidophilus*, *Lacticaseibacillus paracasei* (formerly *L. paracasei*), *Limosilactobacillus fermentum* (formerly *L. fermentum*), and *Bifidobacterium longum*, has been shown to alleviate AD in animal models (Fang et al., 2022; Kim et al., 2019, 2020, 2024; Won et al., 2011). In addition, oral treatment with vaginally derived bacteria, such as *L. crispatus* and *L. gasseri*, has been reported to promote health in organs beyond the gut, including the lungs and reproductive organs (Perez et al., 2025; Tobita et al., 2010). Together, these findings underscore the gut-skin bidirectional communication, commonly described as the gut-skin axis (De Pessemier et al., 2021). However, the precise mechanisms by which probiotics modulate allergic skin inflammation remain to be fully elucidated.

Here, we identified *Lactobacillus crispatus* KBL693, originally isolated from the human vaginal tract, as a promising probiotic candidate that attenuates AD symptoms. Through in vitro assays, we demonstrated its capacity to suppress hallmark immune responses associated with AD. Oral administration of KBL693 alleviated AD-like phenotypes in a mouse model, accompanied by reduced type 2 immune responses as well as an increase in regulatory T cells (Tregs) in gut-draining mesenteric lymph nodes (mLNs). Collectively, these findings support KBL693 as a live biotherapeutic product with potential for AD treatment.

## Materials and Methods

### Bacteria preparation

*Lactobacillus crispatus* KBL693 was previously obtained from vaginal swabs of healthy Korean adults. For in vitro experiments, the bacterium was inoculated into *Lactobacillus* MRS broth (BD Difco, USA) in the presence of L-cysteine (0.05%) at a 1% (v/v) inoculation ratio, and anaerobically cultured at 37℃ for 24 h. The bacterial cultures were subsequently subcultured into fresh MRS broth and incubated under the same conditions for an additional 24 h. Following centrifugation at 3,434 × g, bacterial cells were collected, rinsed two times, and resuspended in phosphate-buffered saline (PBS). For enumeration, bacterial suspensions were incubated with SYTO 9 (10 μM; Invitrogen) for 15 min, then bacterial counts were measured using a CytoFLEX flow cytometer (Beckman Coulter).

For in vivo experiments, the bacterial pellet of *Lactobacillus crispatus* KBL693 was mixed with cryoprotectant agents and lyophilized at -80℃ for 12 h. The lyophilized KBL693 was stored at -20℃ until further use. Bacterial powder was resuspended in PBS with the addition of 0.05% L-cysteine hydrochloride at 5 × 10^7^ or 5 × 10^9^ CFU/ml and mixed for 30 min.

### Cell lines

RBL-2H3 (rat basophilic leukemia, ATCC) and EL4 (mouse T lymphoblast, ATCC) cell lines were used to study mast cell degranulation and type 2 immune cytokine production, respectively. RBL-2H3 cells were grown in DMEM (Gibco) with the addition of 10% FBS (Welgene), 1× NEAA (Gibco), 0.1% sodium bicarbonate (Gibco), penicillin and streptomycin (P/S; 100 U/ml and 100 μg/ml, respectively). EL4 cells were grown in DMEM (Gibco) containing 10% FBS (Welgene), P/S (100 U/ml and 100 μg/ml, respectively). All culture procedures were carried out at 37℃ with 5% CO_2_.

### In vitro mast cell degranulation and IL-4 measurement

For mast cell degranulation, RBL-2H3 seeding was performed in flat-bottomed 96-well plates at 2 × 10^5^ cells/well and cultured in the presence of 0.5 μg/ml anti-DNP IgE (Sigma-Aldrich) for 24 h at 37℃ with 5% CO_2_. After washing twice with 200 μl Siraganian buffer (BioSolution), bacteria were added at a 1:100 cell-to-bacteria ratio and incubated for 20 min. As a positive control, quercetin (40 μM; Sigma-Aldrich) was included. Degranulation was induced by adding 10 μg/ml DNP-HSA (Sigma-Aldrich) for 1 h. Supernatants were harvested, filtered, and assayed for β-hexosaminidase activity. For the assay, 50 μl of each sample was mixed with the equal volume of 3.5 mg/ml p-nitrophenyl-N-acetyl-β-glucosaminide (PNAG; Sigma-Aldrich) in citrate buffer (pH 4.5) and incubated for 2 h at 37℃. Adding 50 μl sodium bicarbonate buffer (pH 10) terminated the reaction. Measurements of absorbance at 405 nm were carried out with a microplate spectrophotometer (Tecan), and results were normalized to the vehicle control group (100%).

For IL-4 measurement, EL4 cells were seeded in flat-bottomed 96-well plates (2 × 10^4^ cells/well) and maintained for 24 h. Bacteria were then added at a 1:100 cell-to-bacteria ratio with PMA and ionomycin (40 ng/ml and 2 μg/ml, respectively). Supernatants were harvested, filtered, and assayed using an ELISA kit (BD Bioscience) following kit protocol.

### Animals and experimental design

NC/Nga mice (6 weeks, female) were provided from Japan SLC. The facility operated on a 12-h alternating light and dark schedule, and environmental conditions were regulated at 20–24℃ and 30–70% humidity. Autoclaved tap water and diet were provided ad libitum. Animal studies were conducted in accordance with protocols approved by the Institutional Animal Care and Use Committee (IACUC) of Seoul National University, Republic of Korea. After one week of acclimation, mice were shaved using clippers and cream.

A DNCB-induced AD model was used to induce AD symptoms (Kim et al., 2018; Scott et al., 2002). DNCB solution (1%) was freshly prepared in a 3:1 mixture of acetone and olive oil and 200 μl was topically applied twice a week for 3 weeks. Subsequently, 0.4% DNCB solution, prepared in the same way, was applied until sacrifice. KBL693 was orally administered daily during the 0.4% DNCB period. A dose of 1 × 10^7^ CFU/mouse was used for a marker analysis cohort, whereas 1 × 10^9^ CFU/mouse was used for the immune cell analysis cohort. On day 44, all mice were sacrificed and plasma and skin tissues were collected from the marker analysis cohort, and mLNs were collected from the immune cell analysis cohort.

### Dermatitis scoring and dorsal thickness measurement

Dermatitis severity was assessed using a modified method described previously (Matsuda, 1997). In brief, erythema, edema, dryness, and abrasion of the DNCB-applied dorsal skin were each evaluated on a scale of 0–3, yielding a total score of 0–12. Dorsal thickness was measured with calipers on the DNCB-applied area.

### Protein preparation and enzyme-linked immunosorbent assay (ELISA)

Skin tissues were chopped with scissors and processed using RIPA buffer (Thermo Fisher Scientific), into which protease and phosphatase inhibitors (Thermo Fisher Scientific) were incorporated, and a steel bead (QIAGEN) using the TissueLyser II (30 Hz, 5 min, twice; QIAGEN). The supernatant obtained after centrifugation was used as the sample for protein marker measurement. IL-4, IL-5, IL-13, and IL-17 were quantified using ELISA kits (BD Bioscience for IL-4 and IL-5; R&D Systems for IL-13 and IL-17), following the protocols provided by the manufacturers. Plasma IgE was quantified using an ELISA kit (BD Bioscience) under the recommended protocol.

### RNA extraction and quantitative PCR

RNA was purified from skin tissue using a kit (iNtRON Biotechnology). In brief, skin tissues were processed in lysis buffer with a 5 mm stainless steel bead using the TissueLyser II (30 Hz, 5 min, twice). The remaining steps were performed following the kit manual. Complementary DNA (cDNA) synthesis was performed with a cDNA kit (Applied Biosystems) and a thermal cycler (Applied Biosystems). Quantitative PCR was carried out using Power SYBR green reagents (Applied Biosystems) using a QuantStudio 5 instrument (Applied Biosystems). Data analysis employed the 2^-ΔΔCt^ method, with *Gapdh* as the reference. Primers were designed as follows:

*Gapdh* F: 5’-ATTGTCAGCAATGCATCCTG-3’, *Gapdh* R: 5’-ATGGACTGTGGTCATGAGCC-3’

*Foxp3* F: 5’-AGAAGCTGGGAGCTATGCAG-3’, *Foxp3* R: 5’-GCTACGATGCAGCAAGAGC-3’

### Preparation of mesenteric lymph nodes and flow cytometry

Mesenteric lymph nodes (mLNs) were excised, minced, and filtered using a 40 μm strainer (Celltrix) to generate single-cell suspensions. Cells were stained with live/dead viability dye (Tonbo Biosciences) for 30 min at 4℃, after which Fc receptors were blocked using anti-CD16/CD32 (clone 93, Invitrogen) for 5 min at 4℃. Subsequently, cells were subjected to a surface antibody cocktail for 30 min at 4℃. The antibody cocktail included anti-CD4^-^FITC (clone RM4-5, Cat. No. 100510, Biolegend), anti-TCRβ-PE-Cy7 (clone H57-597, Cat. No. 25-5961-82, eBioscience), and anti-Nrp-1-PE (clone 3E12, Cat. No. 145204, Biolegend). Foxp3 staining was conducted after fixation and permeabilization with the Transcription Factor Staining Buffer Set (Invitrogen) for 20 min at 4℃. Foxp3 intranuclear staining was performed with anti-Foxp3-PerCP-Cy5.5 (clone FJK-16s, Cat. No. 45-5773-82, eBioscience) for 1 h at 4℃. Cell acquisition was performed on a FACSVerse flow cytometer (BD Biosciences). FlowJo software (Tree Star) was used for data analysis. The gating scheme is presented in Fig. S3.

### Statistical analysis

Results are reported as Mean ± SEM. Statistical analyses and graphing were carried out using GraphPad Prism. Before pairwise comparisons, Shapiro-Wilk test and F-test were applied to verify whether the dataset satisfied normal distribution and equal variance. When the conditions were met, Student’s *t*-test was applied, otherwise the Mann-Whitney U test was employed. Two-way ANOVA was used for time-course data and violations of sphericity were corrected using the Greenhouse-Geisser method. A threshold of *p* value below 0.05 was considered statistically significant.

## Results

### In vitro activity of KBL693 in suppressing type 2 immunity

**Fig. 1.**
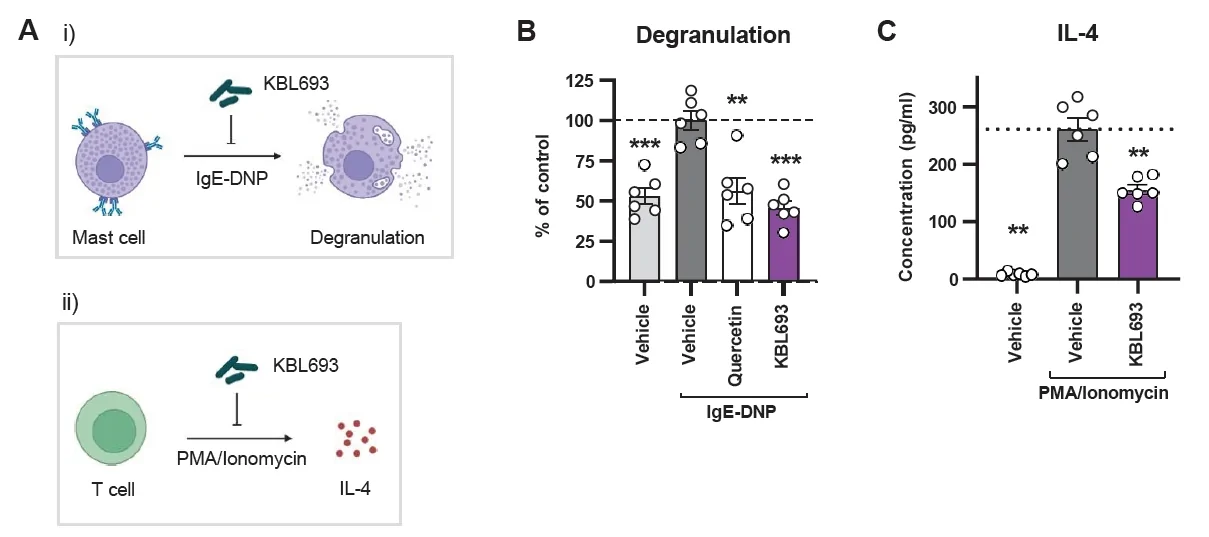
In vitro activity of *Lactobacillus crispatus* KBL693 in suppressing mast cell degranulation and IL-4 production. (A) A schematic overview. (B) Mast cell degranulation using RBL-2H3 basophilic cells after IgE-DNP stimulation. (C) IL-4 production by PMA/ionomycin-stimulated EL4 lymphoblasts. Data are shown as the Mean ± SEM (*n* = 6/group). Statistical significance was determined by Student’s *t*-test (B) and Mann-Whitney U test (C) compared with vehicle group (^**^, *p* < 0.01; ^***^, *p* < 0.001).

Given the involvement of mast cells and Th2 cytokines in atopic dermatitis (AD) pathogenesis, we examined whether *Lactobacillus crispatus* KBL693 could attenuate mast cell degranulation and IL-4 production in vitro (Fig. 1A). Mast cell degranulation was assessed in RBL-2H3 with an IgE-DNP stimulation system. KBL693 significantly suppressed degranulation (45.6 ± 4.15% in KBL693-treated group vs 100 ± 5.71% in vehicle group, *p* < 0.001, *n* = 6; Fig. 1B), reaching levels comparable to the non-stimulated controls. In parallel, IL-4 secretion was measured in PMA/ionomycin-stimulated EL4 cells, where KBL693 markedly reduced IL-4 production (154.6 ± 8.37 pg/ml vs 260.5 ± 19.55 pg/ml, *p* = 0.002, *n* = 6; Fig. 1C).

### Effect of KBL693 on DNCB-induced AD symptoms

**Fig. 2.**
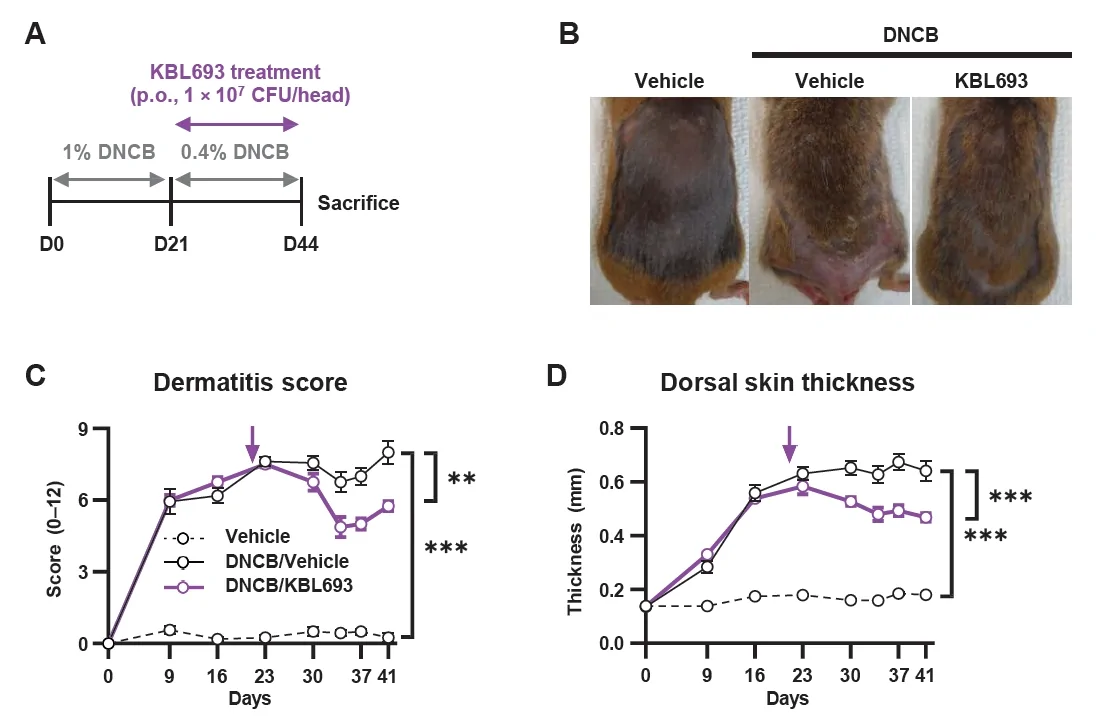
Effect of KBL693 on DNCB-induced AD symptoms. (A) A schematic of the experimental design. (B) Representative images of mice. (C) Time-course of dermatitis score. (D) Time-course of dorsal skin thickness. The time point for initiation of KBL693 treatment is indicated by a purple arrow (C, D). Data are shown as the Mean ± SEM (*n* = 8/group). Statistical significance was determined by two-way ANOVA compared with DNCB/vehicle group (^**^, *p* < 0.01; ^***^, *p* < 0.001).

To evaluate the in vivo efficacy of KBL693, we orally administered it to a mouse model of DNCB-induced AD for 23 days (Fig. 2A). KBL693 treatment significantly reduced dermatitis score (*p* = 0.003 in two-way ANOVA; Fig. 2B and 2C). In addition, each individual criterion of the score was also significantly decreased (Fig. S1A–S1D): erythema (*p* = 0.045), edema (*p* = 0.028), dryness (*p* = 0.008), and abrasion (*p* = 0.041). Dorsal skin thickness also exhibited a sustained reduction beginning at the time of KBL693 administration (*p* < 0.001 in two-way ANOVA; Fig. 2D).

### Effects of KBL693 on type 2 immune biomarkers

**Fig. 3.**
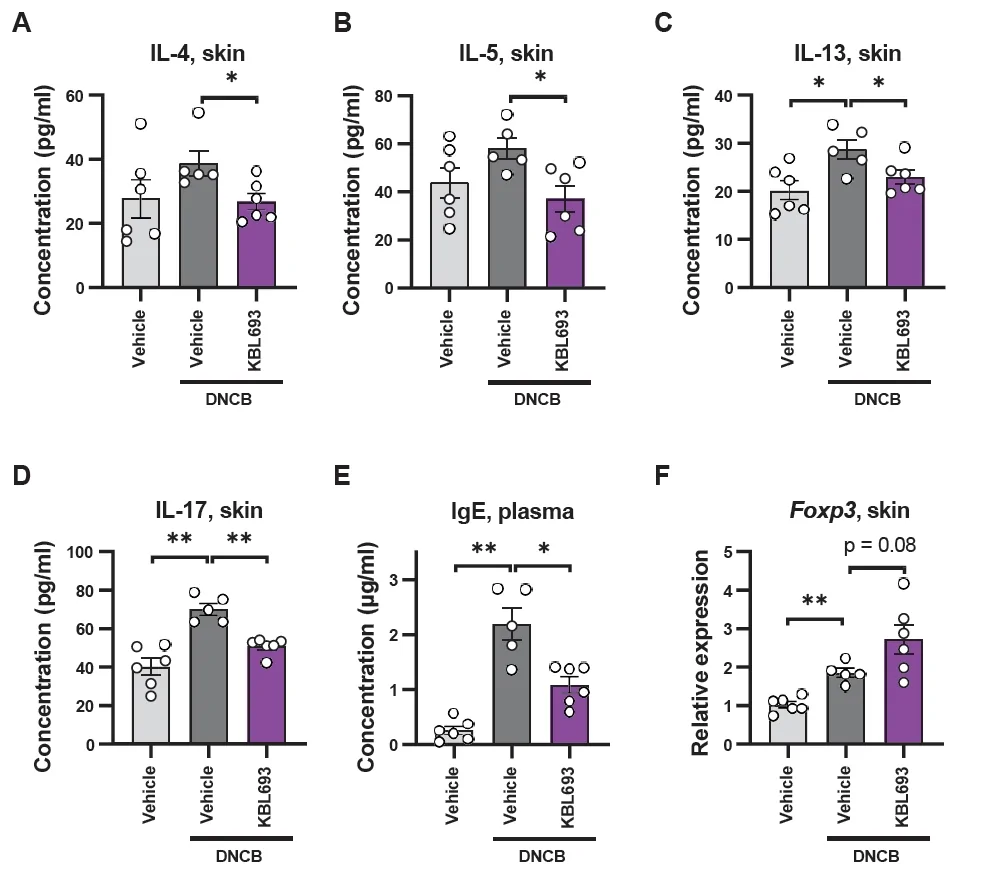
Effects of KBL693 on type 2 immune biomarkers. (A–D) Cytokine levels in skin tissue, including IL-4 (A), IL-5 (B), IL-13 (C), and IL-17 (D). (E) IgE levels in plasma. (F) Relative expression of *Foxp3* in skin tissue. Data are shown as the Mean ± SEM (*n* = 5–6/group). Statistical significance was determined by Student’s *t*-test (B, C) and Mann-Whitney U test (A, D–F) compared with DNCB/vehicle group (^*^, *p* < 0.05; ^**^, *p* < 0.01; ^***^, *p* < 0.001).

Given the phenotypic improvements observed in vivo, we next examined molecular markers associated with allergic responses, which are intimately connected with AD (Akdis et al., 2020). Skin protein levels of Th2 cytokines, including IL-4, IL-5, and IL-13, were significantly reduced (26.8 ± 2.53 pg/ml vs 38.8 ± 3.94 pg/ml in vehicle, *p* = 0.03, 37.1 ± 5.58 pg/ml vs 58.3 ± 4.39 pg/ml, *p* = 0.02, and 23.0 ± 1.44 pg/ml vs 28.7 ± 2.02 pg/ml, *p* = 0.04, respectively; Fig. 3A–3C) and KBL693 also markedly suppressed the Th17 cytokine IL-17 (50.8 ± 1.8 pg/ml vs 69.9 ± 3.1 pg/ml, *p* = 0.004; Fig. 3D). In addition, plasma IgE levels were significantly reduced by KBL693 treatment (1.1 ± 0.14 μg/ml vs 2.2 ± 0.29 μg/ml, *p* = 0.03; Fig. 3E), while *Foxp3* gene expression in skin tissue showed a trend toward increase with KBL693 treatment (2.72 ± 0.38 vs 1.84 ± 0.11, *p* = 0.08; Fig. 3F).

### Treg expansion by KBL693

**Fig. 4.**
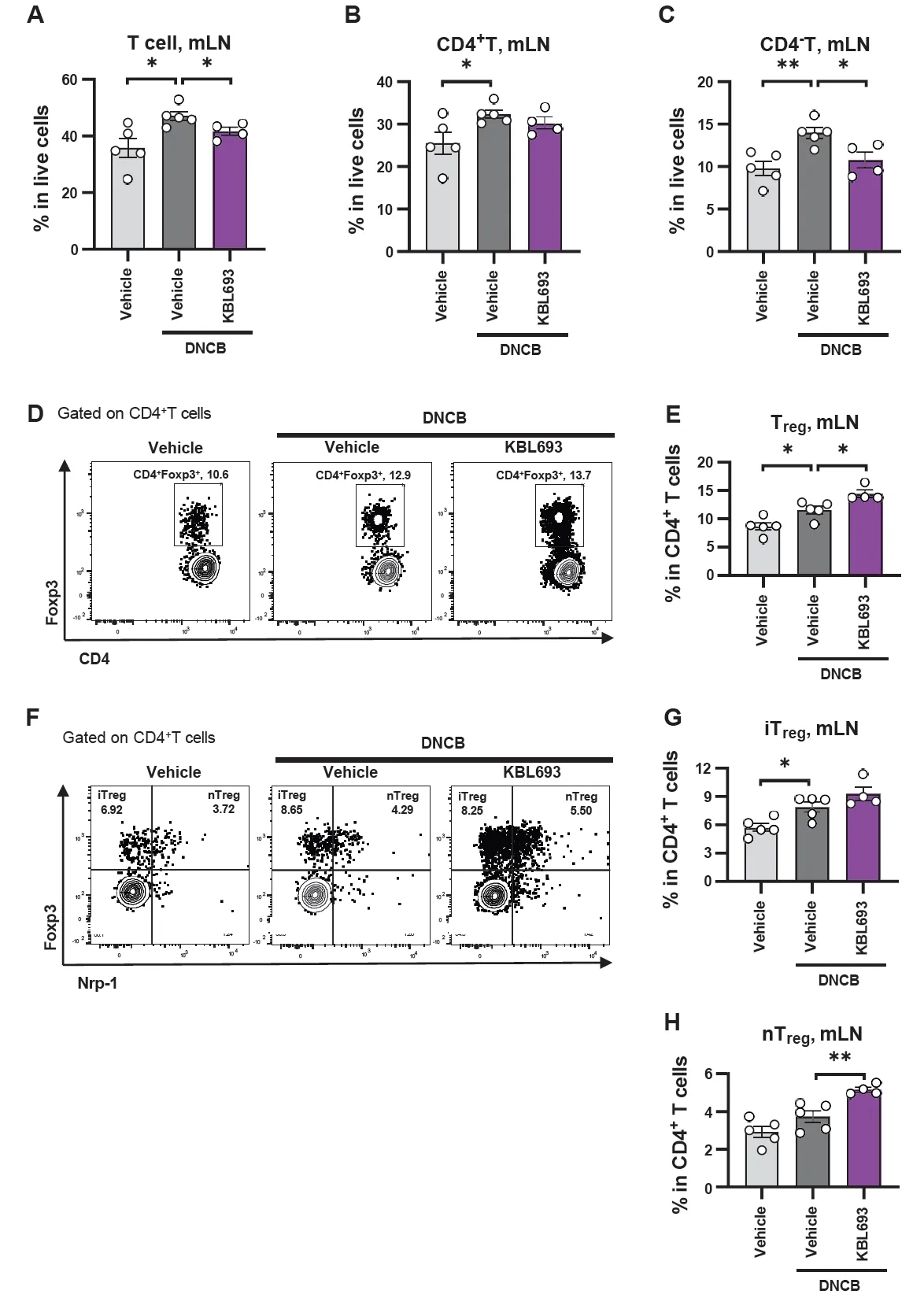
Effect of KBL693 on regulatory T cell increase in mesenteric lymph nodes. (A–C, E, G, H) Proportions of immune cells, including total T cell (A), CD4^+^ T cells (B), CD4^-^ T cells (C), Tregs (E), iTregs (G), and nTregs (H). (D, F) Representative flow cytometry plots of Tregs (D) and their subtypes (F). Data are shown as the Mean ± SEM (*n* = 4–5/group). Statistical significance was determined by Student’s *t*-test (A–C, G, H) and Mann-Whitney U test (E) compared with DNCB/vehicle group (^*^, *p* < 0.05; ^**^, *p* < 0.01).

Given that KBL693 was administered orally, we analyzed immune cell populations in the mesenteric lymph nodes (mLNs), adjacent to the intestine. KBL693 treatment significantly reduced the overall T cell population (41.6 ± 1.33% vs 47.0 ± 1.62%, *p* = 0.02; Fig. 4A). Among the subtypes, CD4^+^ T cell proportions were not affected (30.2 ± 1.38% vs 32.3 ± 0.98%, *p* = 0.23; Fig. 4B), whereas CD4^-^ T cells were significantly decreased by KBL693 (10.7 ± 1.85% vs 14.0 ± 0.66%, *p* = 0.02; Fig. 4C). Within the CD4^+^ T cell, regulatory T cells (Tregs) were significantly increased following KBL693 administration (14.4 ± 0.64% vs 11.5 ± 0.69%, *p* = 0.02; Fig. 4D and 4E). Further subdivision of Tregs based on neuropilin-1 (Nrp-1) expression revealed that induced Tregs (iTregs), which differentiate peripherally in the gut (Yadav et al., 2012), were not altered (9.27 ± 0.72% vs 7.84 ± 0.51%, *p* = 0.14; Fig. 4F–4G), whereas natural Tregs (nTregs), derived from the thymus and circulating in the periphery, were significantly increased by KBL693 (5.14 ± 0.13% vs 3.72 ± 0.31%, *p* = 0.007; Fig. 4F and 4H). For absolute cell numbers, total cells in the mLN were increased by KBL693 (3.13 × 10^6^ ± 1.72 × 10^5^ cells vs 2.18 × 10^6^ ± 3.06 × 10^5^ cells, *p* = 0.04; Fig. S2A), whereas the counts of T, CD4^+^ T, and CD4^-^ T cells remained unchanged (Fig. S2B–S2D). By contrast, the counts of Tregs, iTregs, and nTregs were all significantly expanded following treatment with KBL693 (1.14 × 10^5^ ± 5.05 × 10^3^ cells vs 6.88 × 10^4^ ± 8.28 × 10^3^ cells, *p* = 0.003, 7.32 × 10^4^ ± 2.97 × 10^3^ cells vs 4.64 × 10^4^ ± 5.07 × 10^3^ cells, *p* = 0.004, and 4.13 × 10^4^ ± 3.52 × 10^3^ cells vs 2.26 × 10^4^ ± 3.53 × 10^3^ cells, *p* = 0.008, respectively; Fig. S2E–S2G).

## Discussion

Atopic dermatitis (AD) is a chronic inflammatory skin disease with increasing prevalence worldwide. In this study, we performed in vitro assays to investigate whether KBL693 could reduce mast cell degranulation and IL-4 secretion, which are main effector processes in allergic responses underlying AD (Akdis et al., 2020). As shown in Fig. 1, *Lactobacillus crispatus* KBL693 significantly inhibited mast cell degranulation and IL-4 production. Given that IL-4 drives IgE class switching and elevated IgE enhances mast cell sensitivity to allergen (Pène et al., 1988), these findings suggest that KBL693 may attenuate allergic responses by targeting multiple steps in the pathogenic cascade of AD. These observations prompted us to further evaluate the in vivo efficacy of KBL693 in an animal model of AD.

To determine whether the therapeutic effects of KBL693 observed in vitro could be translated in vivo, we assessed its efficacy in a DNCB-induced mouse model of AD. As shown in Fig. 2, DNCB application induced AD-like symptoms, including increased dermatitis scores and epidermal thickness, providing a basis for evaluating the efficacy of KBL693. KBL693 significantly alleviated dermatitis score as well as each of its four sub-indices (erythema, edema, dryness, and abrasion), suggesting that its benefits may extend to a broad range of patient subgroups. In particular, the reduction of skin thickness implies that KBL693 can mitigate the repetitive itch-scratch cycle commonly observed in patients with chronic AD (Waldman et al., 2018). Moreover, therapeutic efficacy was evident even when treatment was initiated after disease establishment, underscoring the potential of KBL693 as a viable therapeutic intervention rather than merely a preventive strategy. Together, these findings highlight that KBL693 alleviates DNCB-induced AD-like symptoms, supporting its therapeutic potential for AD.

Given that KBL693 exhibited immunomodulatory potential in vitro, we investigated whether it could also modulate type 2 immune responses in vivo by assessing relevant cytokine markers. As shown in Fig. 3, the suppression of degranulation and IL-4 secretion observed in vitro was consistent with the in vivo findings, indicating that KBL693 can attenuate allergic responses through multiple mechanisms. Considering that IL-5 and IL-13 are key factors of eosinophil recruitment and tissue remodeling (Akdis et al., 2020; Fania et al., 2022), KBL693 may exert an amplified impact on suppressing allergic inflammation by attenuating these cytokines in addition to IL-4. In the acute phase of AD, Th2 cells are primarily responsible for allergic responses, whereas in the chronic phase, additional immune pathways, including Th17, become involved to sustain skin inflammation and amplify the repetitive itch-scratch cycle of keratinocytes, ultimately contributing to epidermal thickening (Tsoi et al., 2020; Waldman et al., 2018). Importantly, KBL693 lowered IL-17 levels in addition to Th2 cytokines, suggesting that it may alleviate allergic responses not only during the initial pathogenic phase of AD but also in chronic phase characterized by sustained inflammation. Collectively, the suppression of type 2 cytokines together with the reduction of IL-17 strengthens the evidence that KBL693 mitigates allergic inflammation through both acute and chronic immune pathways.

Regulatory T cells (Tregs) represent a key mechanism for reducing excessive inflammation (Sakaguchi et al., 2020), and several probiotics have been reported to exert their beneficial effects through Treg modulation (Kim et al., 2018, 2020). Consistent with this notion, we observed a trend toward increased *Foxp3* gene expression in skin tissue following KBL693 treatment (Fig. 3F), suggesting that enhanced Treg activity may contribute to the attenuation of Th2- and Th17-mediated inflammation. Given that KBL693 was administered orally and alleviated cutaneous lesions, these observations raise the possibility of a gut-skin axis mediating immune regulation. Consistent with this concept, fecal microbiota transplantation suppresses cutaneous allergic inflammation (Kim et al., 2021), whereas cutaneous inflammation can, in turn, perturb intestinal immunity (Pinget et al., 2022), indicating bidirectional gut-skin crosstalk. Accordingly, we profiled gut-draining mesenteric lymph nodes (mLNs) as a key site where intestinal CD103^+^ dendritic cells can induce Tregs (Coombes et al., 2007; Fig. 4). KBL693 treatment reduced total T cell proportions and CD4^-^TCRβ^+^ cells, presumed to represent CD8^+^ T cells, indicating an overall dampening of immune activation. Moreover, Tregs were increased in the mLNs, providing additional evidence that KBL693 enhances immunoregulatory capacity at the gut level.

Tregs can arise as natural Tregs (nTregs or thymic Tregs, tTregs) generated in the thymus or as induced Tregs (iTregs, or peripheral Tregs, pTregs) that differentiate in the periphery upon antigen encounter in mucosal areas. These subsets can be distinguished by the Nrp-1 marker (Yadav et al., 2012). Our data indicate that KBL693 increased both the proportion and absolute number of Tregs in the mLNs (Fig. 4). Notably, nTregs were consistently expanded at both the proportional and absolute levels, supporting the notion that KBL693 enhances thymus-derived Treg differentiation. Previous reports demonstrated gut microbiota can influence thymic function (Balcells et al., 2022; Nakajima et al., 2014), raising the possibility that KBL693 boosts Treg differentiation to increase circulating nTregs. Expansion of this circulating pool may in turn facilitate the accumulation of Tregs in the skin and mLN, thereby reinforcing local immune suppression in AD. Although iTregs did not increase in proportion, their absolute counts were significantly elevated, suggesting that peripheral induction may also facilitate the overall expansion of the Tregs. Collectively, these findings indicate that KBL693 enhances Treg abundance in the mLNs and supports increased Treg activity (*Foxp3* gene expression) in the skin, providing a plausible mechanism by which this strain suppresses allergic responses in AD.

These findings have several implications. First, because KBL693 exerted direct immunomodulatory effects in vitro, it is plausible that the strain stimulates host pattern recognition receptors, such as those recognizing flagellin, peptidoglycan, or lipopolysaccharide (Cho et al., 2025; Jordan et al., 2023; Seo et al., 2020). Identifying the specific bacterial component responsible for this activity would greatly broaden our understanding of the strain’s mechanism. Second, given the suggested gut-skin axis, it remains to be determined whether the effects of KBL693 are mediated by the gut-to-skin immune cell migration or by bacterial components (or metabolites) that circulate systemically and directly act on skin tissue. Elucidating the mechanisms underlying this gut-skin axis may not only advance our understanding of inter-organ immune communication but also facilitate the discovery of novel biomarkers for allergic diseases. Third, KBL693 is a strain of *Lactobacillus crispatus*, a species most commonly associated with the vaginal microbiota rather than the adult gut (Ravel et al., 2011). Coupled with evidence that maternal vaginal bacteria are transmitted at birth to help establish the early-life gut microbiota and are associated with immune tolerance (McCauley et al., 2022), our results suggest that vaginally derived strains delivered orally can influence systemic immunity. This perspective aligns with our mLN findings and provides a plausible route by which a vaginal-origin probiotic might affect cutaneous inflammation via the gut-skin axis. While skin-draining lymph nodes (axillary, brachial, and inguinal) are central to cutaneous immunity, our study was limited to mLNs. Further studies incorporating immunophenotyping of skin-draining lymph nodes should help clarify KBL693’s mechanism.

In conclusion, we demonstrated that the vaginal strain *Lactobacillus crispatus* KBL693 alleviates hallmark features of AD. Notably, KBL693 increased the frequency of nTregs in mLNs, thereby suppressing immune activation, including allergic responses. These findings support the potential of KBL693 as a strain-specific probiotic candidate for AD therapy.

## Supplementary Information

Fig. S1. Effect of KBL693 on individual dermatitis indices in the DNCB-induced mouse model. (A–D) Time-course of erythema (A), edema (B), dryness (C), and abrasion (D), the time point for initiation of KBL693 treatment is indicated by a purple arrow. Data are shown as the Mean ± SEM (*n* = 8/group). Statistical significance was determined by two-way ANOVA compared with DNCB/vehicle group (^*^, *p* < 0.05; ^**^, *p* < 0.01; ^***^, *p* < 0.001).

Fig. S2. Increase of regulatory T cell counts in mesenteric lymph nodes by KBL693. (A–C, E, G, H) Absolute immune cell numbers, including total cells (A), T cells (B) CD4^+^ T cells (C), CD4^-^ T cells (D), Tregs (E), iTregs (F), and nTregs (G). Data are shown as the Mean ± SEM (*n* = 4-5/group). Statistical significance was determined by Student’s *t*-test (A, C–G) and Mann-Whitney U test (B) compared with DNCB/vehicle group (^*^, *p* < 0.05; ^**^, *p* < 0.01).

Fig. S3. Gating strategy.
