# Supplementary material for: Lactobacillus crispatus KBL693 alleviates atopic dermatitis symptoms through immune modulation
Source: J Microbiol. 2025 Oct 31;63(10):e2509005. doi: 10.71150/jm.2509005 (PMC13577048; doi:10.71150/jm.2509005)
Supplement: Supplementary file 1 [file jm-2509005-Supplementary-Fig-S1.pdf]

Fig. S1

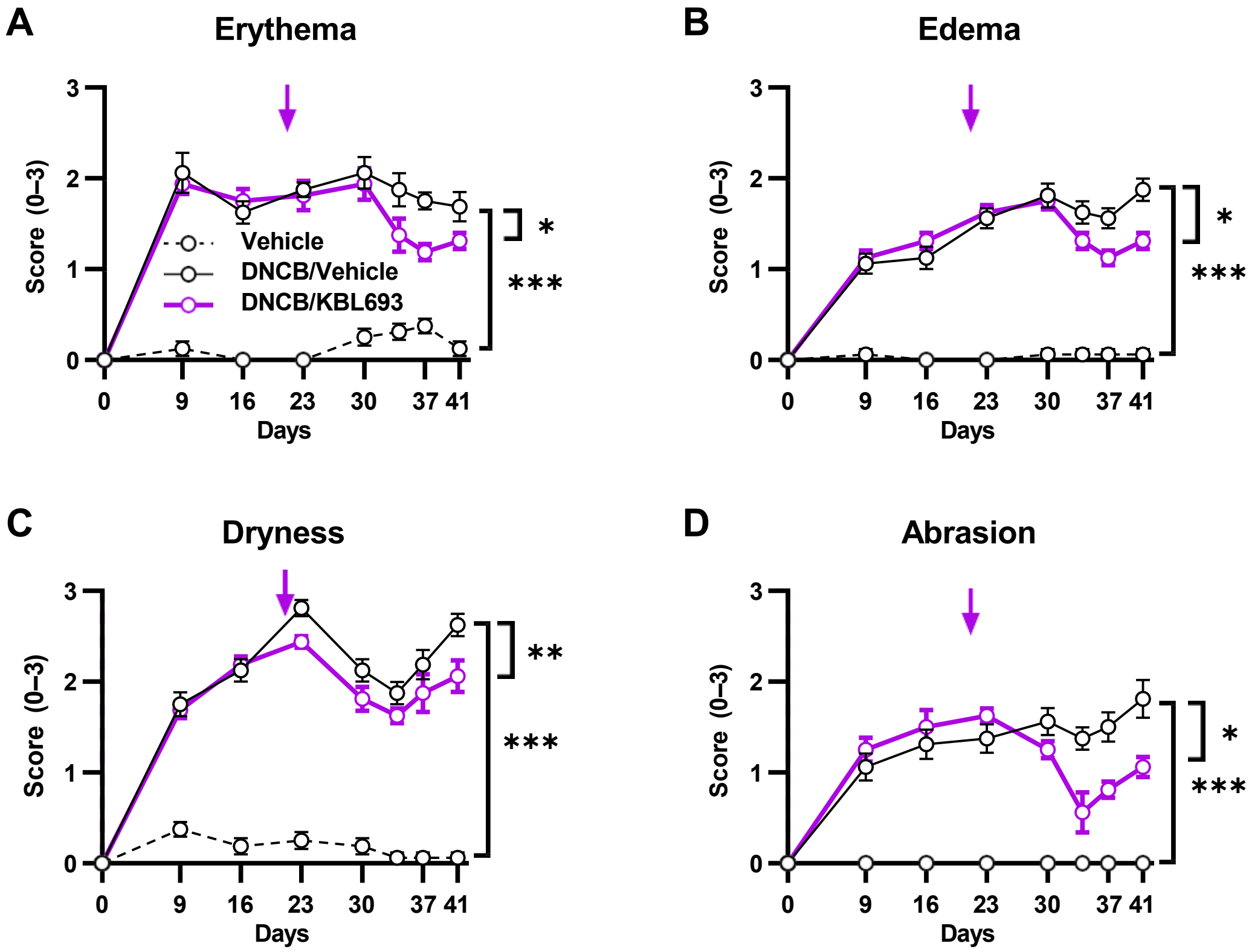
A
Erythema
Score (0–3)
Vehicle
DNCB/Vehicle
DNCB/KBL693
*
***
Days
0
9
16
23
30
37
41
B
Edema
C
Dryness
**
D
Abrasion
