# Supplementary material for: Lactobacillus crispatus KBL693 alleviates atopic dermatitis symptoms through immune modulation
Source: J Microbiol. 2025 Oct 31;63(10):e2509005. doi: 10.71150/jm.2509005 (PMC13577048; doi:10.71150/jm.2509005)
Supplement: Supplementary file 2 [file jm-2509005-Supplementary-Fig-S2.pdf]

Fig. S2

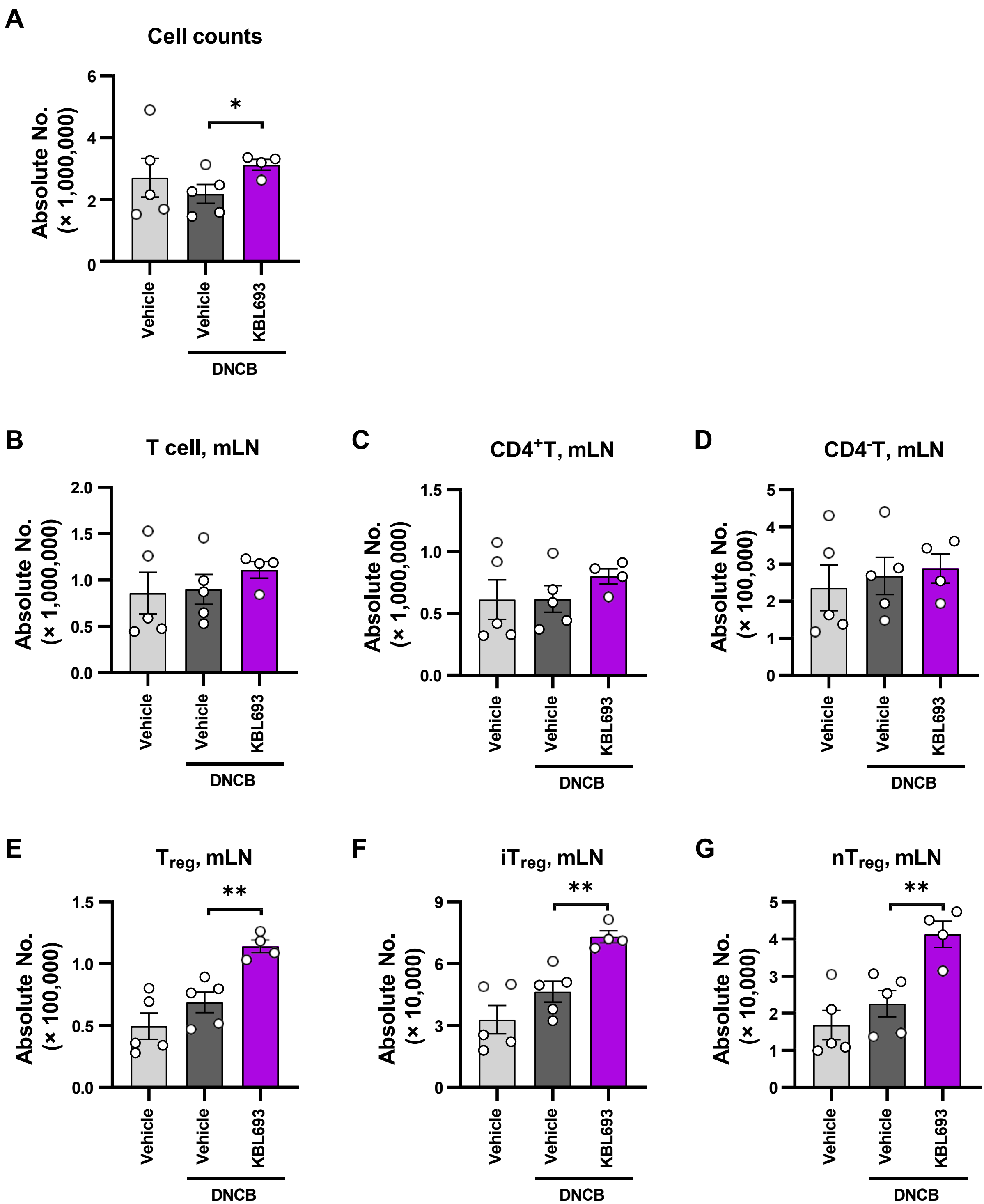
A
Cell counts
Absolute No. (× 1,000,000)
*
Vehicle
Vehicle
KBL693
DNCB
B
T cell, mLN
Absolute No. (× 1,000,000)
C
CD4+T, mLN
Absolute No. (× 1,000,000)
D
CD4-T, mLN
Absolute No. (× 100,000)
E
Treg, mLN
Absolute No. (× 100,000)
**
F
iTreg, mLN
Absolute No. (× 10,000)
**
G
nTreg, mLN
Absolute No. (× 10,000)
**
