# Supplementary material for: Lactobacillus crispatus KBL693 alleviates atopic dermatitis symptoms through immune modulation
Source: J Microbiol. 2025 Oct 31;63(10):e2509005. doi: 10.71150/jm.2509005 (PMC13577048; doi:10.71150/jm.2509005)
Supplement: Supplementary file 3 [file jm-2509005-Supplementary-Fig-S3.pdf]

Fig. S3

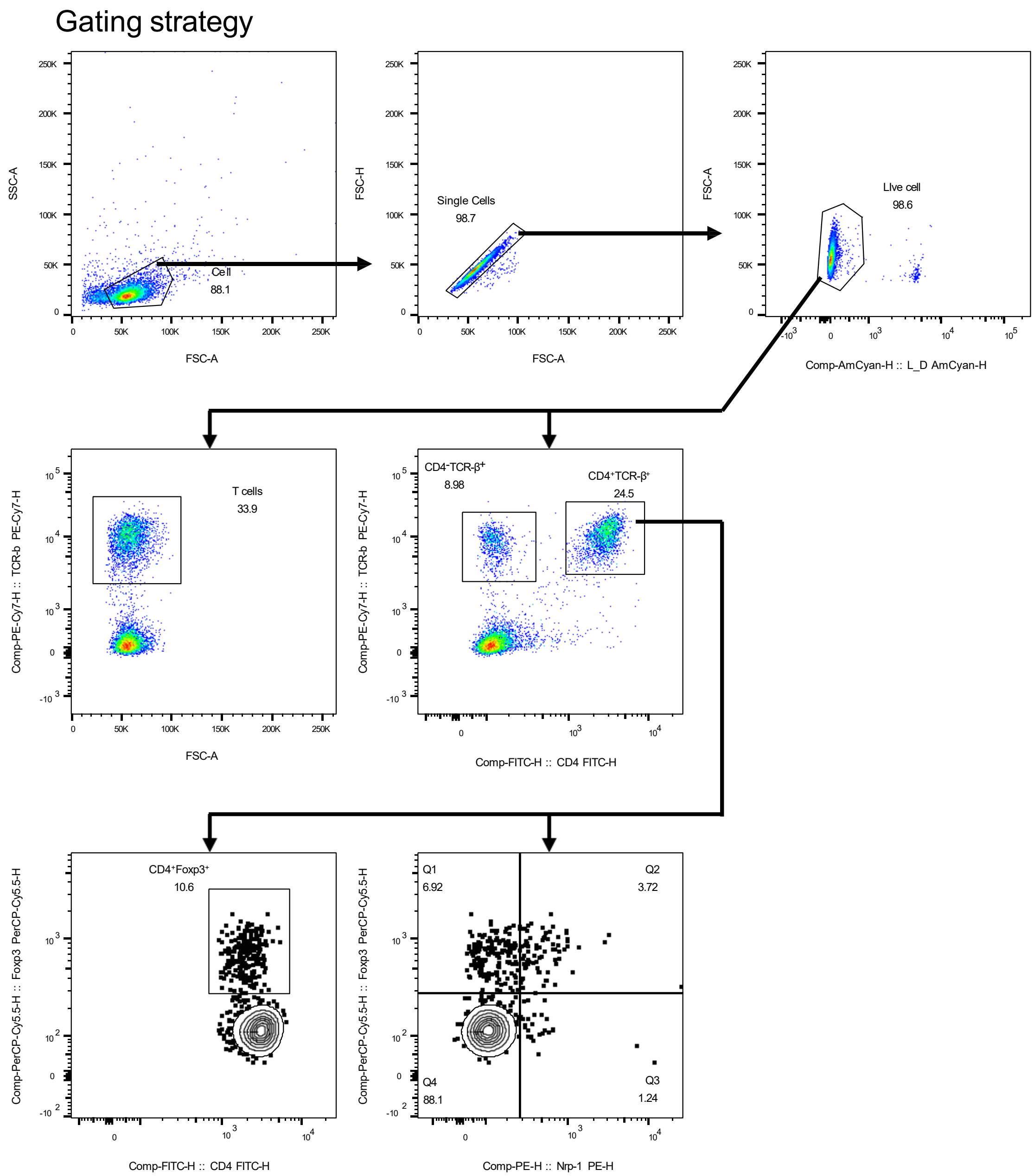
Gating strategy
SSC-A
FSC-A
Cell
88.1
FSC-H
Single Cells
98.7
FSC-A
LIve cell
98.6
Comp-AmCyan-H :: L_D AmCyan-H
T cells
33.9
Comp-PE-Cy7-H :: TCR-b PE-Cy7-H
FSC-A
CD4⁻TCR-β⁺
8.98
CD4⁺TCR-β⁺
24.5
Comp-FITC-H :: CD4 FITC-H
CD4⁺Foxp3⁺
10.6
Comp-PerCP-Cy5.5-H :: Foxp3 PerCP-Cy5.5-H
Comp-FITC-H :: CD4 FITC-H
Q1
6.92
Q2
3.72
Q4
88.1
Q3
1.24
Comp-PE-H :: Nrp-1 PE-H
